# 1015. Case Study. Staged Excision and Skin Grafting for Perineoscrotal Hidradenitis Suppurativa: A Burn Center Case Series

**DOI:** 10.1093/jbcr/irag033.203

**Published:** 2026-04-06

**Authors:** Kyung Yoon, Vaisny Balamurali, Tara Ranjbar, Hilla Katz-Lichtenstein, Michael L Cooper

**Affiliations:** Northwell, Marietta, GA; Ross University School of Medicine, Staten Island, NY; Northwell, Staten Island, NY; Northwell, Staten Island, NY; Staten Island University Hospital Northwell Health, Staten Island, NY

## Abstract

**Patient Presentation (age range, injury details, relevant history):**

Four male patients, ages 19–63, presented with severe perineoscrotal hidradenitis suppurativa (HS), a chronic, relapsing inflammatory skin disorder. Disease onset ranged from adolescence to adulthood. All patients had previously failed prolonged systemic antibiotic therapy and adalimumab (Humira), with one developing Humira-related cardiomyopathy. Early-onset patients (< 30 years) demonstrated widespread involvement including the perineum, scrotum, thighs, groins, and axillae, while older patients had more localized perineoscrotal disease. Many patients reported prior abscess drainage procedures and significant functional limitations, including pain with ambulation, hygiene difficulties, and interference with work or daily activity.

**Clinical Challenges:**

Perineoscrotal HS presents unique challenges due to constant moisture, bacterial colonization, contamination from urine and stool, and a high risk of recurrent infection. Early-onset disease often spans multiple intertriginous regions, requiring extensive excision and complex reconstruction. Optimal timing, number of staged debridements, and grafting strategies remain poorly defined. Reconstruction must achieve durable wound coverage, minimize graft loss in contaminated fields, and preserve genital and perineal function while preventing recurrence. Severe HS often significantly impacts quality of life, underscoring the importance of an effective surgical approach.

**Management Approach:**

All patients underwent staged scalpel-based wide excision with serial debridement every 3–4 days to remove nodules, sinus tracts, and fibrotic tissue down to healthy fascia. Wounds were irrigated and assessed for granulation tissue formation. Each patient required 3–5 staged debridements prior to reconstruction. Once the wound bed was healthy, delayed split-thickness skin grafting (STSG) was performed, harvested from the thigh and meshed 1:1.5. Bolster dressings and negative-pressure wound therapy were applied to optimize graft adherence. This staged approach aimed to eradicate disease, maximize graft take, and reduce recurrence while minimizing donor site morbidity.

**Outcomes:**

All patients achieved excellent STSG take with no major graft loss. Early-onset patients required longer stays (35–42 days) and more procedures. Older patients had shorter LOS (18–24 days). One patient (age 19) recurred at three years; others remain recurrence-free. One developed Humira-related cardiomyopathy. Overall, staged excision and delayed grafting provided durable reconstruction with preserved function and low complication rates.

**Lessons Learned:**

Early surgical referral is critical. Burn surgery principles—serial debridement, wound optimization, delayed STSG—improve outcomes in severe HS. Medical therapy may fail, emphasizing timely operative intervention. Staged reconstruction provides durable coverage, preserves function, and minimizes short-term complications.

**Applicability to Practice:**

Burn centers are well-positioned to manage severe perineoscrotal HS. Applying staged excision, wound bed preparation, and delayed STSG allows durable, reproducible reconstruction, reduces recurrence, shortens hospital stay, and optimizes functional and cosmetic outcomes.

